# Soil Types Effect on Grape and Wine Composition in Helan Mountain Area of Ningxia

**DOI:** 10.1371/journal.pone.0116690

**Published:** 2015-02-23

**Authors:** Rui Wang, Quan Sun, Qingrui Chang

**Affiliations:** 1 College of Resource and Environment, Northwest A&F University, Yangling, Shaanxi, 712100, P.R. China; 2 Grape and Wine Engineering Research Centre of Education Ministry, Ningxia University, Yinchuan, Ningxia, 750021, P.R. China; Colorado State University, UNITED STATES

## Abstract

Different soil types can significantly affect the composition of wine grapes and the final wine product. In this study, the effects of soil types on the composition of *Cabernet Sauvignon* grapes and wine produced in the Helan Mountains were evaluated. Three different representative soil types—aeolian, sierozem and irrigation silting soil were studied. The compositions of grapes and wines were measured, and in addition, the weights of 100-berry samples were determined. The grapes that grown on the aeolian and sierozem soils matured sooner than those grown on the irrigation silting soil. The highest sugar content, total soluble solids content, sugar to acid ratio and anthocyanin content were found in the grapes that grown on the aeolian soil. The wine produced from this soil had improved chroma and tone and higher-quality phenols. The grapes grown on the sierozem soil had the highest total phenol and tannin contents, which affected the wine composition. The grapes grown on the irrigation silting soil had higher acidities, but the remaining indices were lower. In addition, the grapes grown on the aeolian soil resulted in wines with better chroma and aroma. The sierozem soil was beneficial for the formation of wine tannins and phenols and significantly affected the wine composition. The quality of the grapes from the irrigation silting soil was relatively low, resulting in lower-quality wine.

## Introduction

Soil provides the foundation for grape vine growth, providing the necessary water and nutrition [1]. The physical and chemical characteristics of soils, such as soil type, soil structure, soil depth, fertility, temperature and soil moisture, directly affect vine root growth and nutrient absorption [2]. Although wine grapes can adapt to many soil types, grape and wine composition are significantly affected by soil types, which influences the taste of the final product. Wine grapes grown on highly permeable soils and under the same environmental conditions with large diurnal temperature differences have faster photosynthetic rates, higher sugar concentrations, and improved chroma and palate [3,4]. Nutrients are more easily absorbed in slightly alkaline to neutral pH soils, which improves vegetative vine growth and fruit quality [5]. Soil fertility, particularly soil potassium and calcium concentrations, and soil microorganisms can influence the sugar and tannin contents of wine grapes [6,7]. Soils that are rich in P and Ca promote the accumulation of sugar and the formation of aromatics and anthocyanin in grapes [8,9].

The effects of regional characteristics such as soil type, climate and topography on grape phenolic compounds have been widely investigated [10,11]. All aspects of the soil-plant-atmosphere interaction are known to control grape berry composition [12]. Therefore, different soil types can lead to discrepancies in wine composition, even under the same climate conductions [5,12,13–15].

The composition of wine mainly depends on the composition of the grapes, followed by the wine-making technique [16]. The sugar, acid, tannins, anthocyanin and aromatics contents of the grapes and their interactions play key roles in the composition of wine [17]. Phenolic compounds, which mainly consist of anthocyanin, flavonoids, phenolic acids and stilbenes, play an important role in the composition of red grapes and wines [18]. These compounds contribute to the color and palatability of red wines [19,20]. Consequently, grape variety and fermentative and aging conditions affect the composition and content of phenolic compounds in wines. Regarding single-variety wine, the composition and content of phenolic compounds largely depend on the vineyard [21]. In Andalusia (southern Spain), there are more than 22 main wine-producing sub-regions, and each sub-region produces representative wines due to its local soil characteristics [22]. Several studies have investigated the effects of climate, canopy microclimate, soil types, and water status on the accumulation of phenolic compounds in grapes, revealing that most of these factors can affect the accumulation of phenolic compounds [2,23–25]. However, most of these studies focused on the effects of particular factors on phenol accumulation and were generally not concerned with the impacts of soil types. According to Li et al., understanding the impacts of soil types on the phenolic compounds in grapes and wines is necessary for identifying moderate grapevine cultivars and for developing an effective viticulture management program [21]. It is difficult to study the effects of soil types on grape and wine composition because they involve many factors. Currently, the pH, mineral content, and fertility of vineyard soils can be artificially modified to improve the quality of grapes. However, the soil types of a vineyard cannot be easily changed. Therefore, it is important to study the grape and wine composition from different soil types [8,15,21,26,27].

Different soils types have different soil physical structures and mineral nutrient contents. Because aeolian soil has high sand contents, it has highly permeable, low nutrient and plant available water contents (PAWC). Irrigation silting soil has equivalent percentages of clay, silt and sand, which makes it less permeable, with medium levels of PAWC. The structure of sierozem soil is loose and highly permeable, which results in moderate PAWC and fertility with low organic matter and mineral nutrient contents and high Ca concentrations [3,15]. Generally, the most favorable soil types for grape production in this arid region are gravelly and sandy loam soils, and grapes grown in gravelly soils result in better wine quality [3,28].

This study aimed to analyze the differences in wine grapes and wine composition among three soil types in the Helan Mountains. The objective of this research was to evaluate the relationships between soil types and wine grape composition and to provide practical information for adjusting vineyard growing practices and optimizing wine flavor.

## Materials and Methods

### Ethics statement

The study was approved by the Grape and Wine Engineering Research Centre of Education Ministry and was conducted at their experimental station in Ningxia (NW China).

### The study area

The vineyards are located on the Helan Mountain alluvial plain in Ningxia (NW China) (37°43′-39°23′ N, 105°45′-106°47′ E) at an altitude of approximately 1035 m with a cool, semi-arid climate and large diurnal temperature differences. The annual accumulated temperature varies from 3298–3351°C, with abundant sunshine of annual average sunshine 2800~3000 hours and low annual rainfall of 158–200 mm. Early frosts are common on the slopes of the Helan Mountains, and winter temperatures are very low (<-10). Therefore, the grapevines must be covered with soil to prevent injury. Any harvesting delay will reduce the available time for pruning and soil covering. In 2013, three wine-growing areas with distinct soil types were selected: an aeolian soil area (Yuquanying town, 38°23′37.38″N, 106°9′25.14″E), a sierozem area (Qingtongxia city, 38°27′0.28″N, 106°1′31.54″E) and an irrigation silting soil area (Luhua town, 38°34′13.73″N, 106°10′25.06″E). The three regions showing a triangular distribution and direct distance of them are less than 20 km, the altitude, light, rainfall and other climatic conditions are similar. All test points are our school experimental base, in order to understand the difference in quality wines under different soil types, all cultivation and management are the same, unified pruning, fertilization, irrigation and disease prevention. The basic soil physical and chemical parameters from these areas are shown in Table 1. The *Cabernet Sauvignon* grape variety was planted in 2006 in rows orientated north to south and with an individual vine density of 0.5 × 3.0 m.

**Table 1:** Basic physical and chemical parameters of three soil types.

Treatment	Organic matter g kg^-1^	Available N mg kg^-1^	Available P mg kg^-1^	Available K mg kg^-1^	Exchangeable Ca mg kg^-1^	pH	Percentage of each grain size %		
							Clay <0.002 mm	Silt 0.002–0.02 mm	Sand 0.02–2 mm
Aeolian soil	2.74	22.35	18.64	68.44	402.27	8.43	3.45	6.11	90.44
Sierozem	4.46	48.25	32.79	97.27	438.91	8.41	12.65	38.62	48.73
Irrigation silting soil	10.57	76.73	88.92	198.31	415.67	8.40	28.97	40.37	30.66

### Composition analysis of grapes and wines

**Fig 1:**
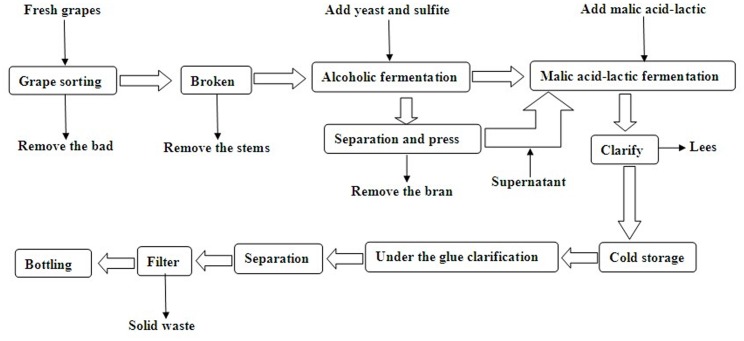
Wine making process schematic.

In 2013, sixty plants were selected from each soil type in the vineyards as the study subjects. At the commencement of veraison (~mid-August), samples were collected weekly over a 8-week period until final harvest, which usually occurred during the first week of October. During the final harvest of each replicate, a random sub-sample of 100 grapes was weighed to determine the average grape weight. The samples were frozen and stored for later analyses, including tannin, anthocyanin, and iron-reactive phenolic content analyses. ^0^Brix was determined using a Brix refractometer (PR32 Atago Co. Ltd., Japan). Reducing sugars were determined using titration with Fehling reagent, and titratable acidity was determined using standardized 0.1 N NaOH (end-point pH 8.2). The pH of the juice was recorded using a Metmorph 702SM automatic neutralizer (Titrino, Herisau, Switzerland) [3,8,15]. Whole grapes from the frozen samples were homogenized and analyzed for their anthocyanin, tannin, and iron-reactive phenolic concentrations, which were determined using spectrophotometry after extraction with ethanol [29,30]. The wine chroma was also measured using spectrophotometry, alcohol and dry extract contents were determined using the wine generic analytical method [21]. In this study, the details of winemaking process refer to Fig. 1.

### Statistical methods

Significant differences among soil types were random determined by methods of one-way ANOVA and two-way analysis of variance (ANOVA) followed by the Student-Newman-Keuls test using the SAS 8.1 software (SAS Institute Inc., Cary, NC, USA) followed by the least significant difference (LSD) test for multiple comparisons among groups. Differences with *p* values of less than 5% (*p*<0.05) were considered statistically significant.

## Results

### 100-berry weight

**Fig 2:**
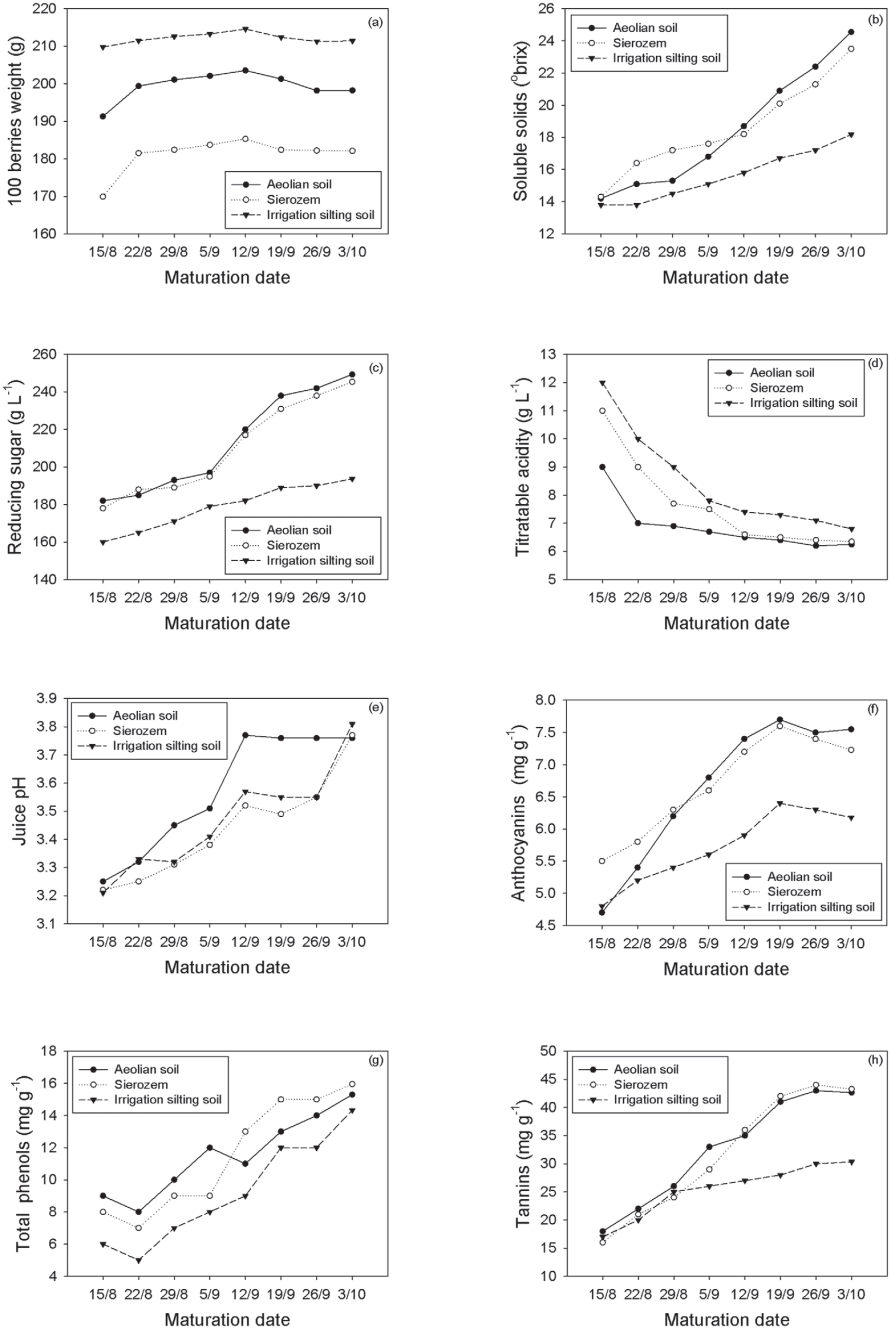
Changes in grape composition.

During the maturation period, the 100-berry weight varied significantly among the different soil types (Fig. 2a). It increased during the early maturation period and stabilized in mid-September. The grapes grown on the aeolian and sierozem soils had loose clusters with relatively lower 100-berry weights relative to the grapes grown on the irrigation silting soils (Table 2). Grapes grown on sierozem soil had the lowest 100-berry weight which was significantly lower than the grapes grown on the aeolian soil. The grapes from the irrigation silting soil vineyards had tight clusters and significantly greater 100-berry weights than the aeolian and sierozem soil sites.

**Table 2:** Analytical composition of the grapes at harvest period on three soil types.

Treatment	Weight of 100 berries g	Soluble solids°brix	Reducing sugar g L^-1^	Titratable acidity g L^-1^	Juice pH	Anthocyanins mg g^-1^	Total phenols mg g^-1^	Tannins mg g^-1^
Aeolian soil	198.23±5.32b	24.55±0.45a	249.35±6.32a	6.25±0.01b	3.76±0.02a	7.55±0.01a	15.30±0.02b	42.65±0.05a
Sierozem	182.10±2.26c	23.50±0.26a	245.40±4.17a	6.35±0.02b	3.77±0.01a	7.23±0.01b	15.95±0.02a	43.25±0.04a
Irrigation silting soil	211.45±3.57a	18.18±1.31b	193.75±2.29b	6.80±0.01a	3.81±0.01a	6.18±0.02c	14.33±0.03c	30.35±0.07b

Note: Multiple comparisons were conducted among different levels of the same factor in one column (p<0.05).

### Total soluble solids and reducing sugar content

Generally, the total soluble solids increased during the maturation period of the grapes (Fig. 2b). During early maturation, the total soluble solids concentrations in the grapes grown on the aeolian soils were significantly lower than those grown on the sierozem soils. However, by mid-maturation, the situation was reversed. Over the entire maturation period, the total soluble solids concentrations from the grapes grown on the irrigation silting soil increased steadily (Fig. 2b), which was significantly lower than the total soluble solids concentrations of the grapes grown on the other soils (Table 2). The grapes harvested on October 3rd from the aeolian soil had the highest soluble solids content, which was not significantly different from that of sierozem soils.

During the first 3 weeks of maturation, the grape reducing sugar content increased steadily in all soil types. However, the accumulation rates increased during the subsequent 2 weeks for the grapes that were grown on the aeolian and sierozem soils (Fig. 2c). The reducing sugar accumulation rates of the grapes grown on the irrigation silting soil steadily increased (Fig. 2c). The reducing sugar contents in the final harvest grapes from the aeolian and sierozem soils were not significantly different. However, these reducing sugar contents were significantly greater than those of the grapes grown on the irrigation silting soils.

### Titratable acidity and pH

The titratable acidity increased steadily during maturation and decreased rapidly during the first 3 weeks (Fig. 2d). The titratable acidities of the grapes grown on the irrigation silting soil, sierozem, and aeolian soils decreased respectively, during the first week. At final harvest, the titratable acidities of the grapes grown on the aeolian and sierozem soils were similar to each other, and they were significantly lower than the values of the grapes grown on the irrigation silting soils.

During the first 5 weeks of maturation, the pH of the grape juice generally increased by 0.10 pH units each week (Fig. 2e). Although the pH of the grapes grown on the aeolian soils stabilized at 3.76 during the remainder of the harvest period, the grapes grown on the other soils stabilized at a pH that was 0.30 pH units lower for 2 weeks before reaching an equivalent level (Table 2) in the final week.

### Sugar to acid ratio

Sugar to acid ratios in the grapes from the aeolian and sierozem soils were generally higher than those in the grapes from the irrigation silting soil. Until September 5th, the values were similar, but they tended to diverge in the final 4 weeks (Table 3). At harvest, the sugar to acid ratio of the wine grapes from the aeolian soil reached 40.16, followed by 38.89 for the grapes from the sierozem soil and 28.38 for the grapes from the irrigation silting soil (Table 3). The sugar to acid ratio is a determinate of grape maturity and indicated that grape maturity occurred in the following order, from earliest to latest: aeolian, sierozem and irrigation silting soil.

**Table 3:** Changes in the sugar to acid ratio during the maturation period.

Treatment	Date							
	15/8	22/8	29/8	5/9	12/9	19/9	26/9	3/10
Aeolian soil	20.22±1.34a	26.43±0.24a	27.97±2.22a	29.40±0.98a	33.85±0.56a	37.19±0.37a	39.03±1.02a	40.16±0.57a
Sierozem	16.18±0.26b	20.89±0.06b	24.55±0.64b	26.00±1.37b	32.88±0.29a	35.54±0.22a	37.19±1.11a	38.89±0.52a
Irrigation silting soil	13.33±0.57c	16.50±1.33c	19.00±0.92c	22.95±1.11c	24.59±1.18b	25.89±1.15b	26.76±0.97b	28.38±1.01b

Note: Multiple comparisons were conducted among different levels of the same factor in one column (p<0.05).

### Analysis of anthocyanin

The anthocyanin contents in the grape during the first 5 weeks of maturation increased before decreasing over the final 2 weeks (Fig. 2f) in all treatments. Throughout the sampling period, the anthocyanin contents of the grapes grown on the aeolian and sierozem soils remained significantly greater than those grown on the irrigation silting soil. At harvest, the anthocyanin content decreased slightly in all treatments. The highest anthocyanin content appeared in the wine grapes from the aeolian soil, which was significantly higher than the contents obtained from those grown on the sierozem soil. The lowest anthocyanin content was observed in the grapes grown on the irrigation silting soil and was significantly lower than the content observed in the grapes grown on the sierozem soil (Fig. 2f).

### Total phenol and tannin contents

During the early stages of the mature period, the total phenol contents in the wine grapes from the sierozem soil were lower than those grown on the aeolian soil (Fig. 2g). After September 12th, berry maturation was accelerated. Thus, the total phenol contents of the wine grapes grown on the three soil types increased rapidly. The grapes from the sierozem soil achieved the fastest rate, surpassing the rates of those grown on the aeolian soil. At harvest, the total phenol contents of the grapes from the sierozem soil reached 15.95 mg g^-1^, which was significantly greater than that of the grapes grown on the aeolian soil. The total phenol contents of the grapes from the irrigation silting soil were the lowest among the treatments and were significantly lower than in the grapes grown on the aeolian soil.

Before August 29th, the tannin contents of the wine grapes from the three soil types were similar. However, after August 29th, the tannin contents of the wine grapes from the sierozem and aeolian soils rapidly increased, reaching a stable value by September 26th. However, the tannin contents of the wine grapes from the irrigation silting soil continued to increase gradually (Fig. 2h). At harvest, the tannin contents of the wine grapes from the sierozem soil reached 43.25 mg g^-1^, followed by 42.65 mg g^-1^ from the aeolian soil. However, the difference between the sierozem and aeolian soils was not significant. The tannin content of the grapes from the irrigation silting soil was only 30.35 mg g^-1^, which was significantly lower than that of the grapes from the other two soil types.

### Effects of soil types on wine composition

According to Table 4, the alcohol contents of the wines made from the grapes grown on the aeolian and sierozem soils were significantly greater than from the grapes grown on the irrigation silting soil. No significant differences occurred regarding the remaining sugar contents, dry extract concentrations, and pH among the wines produced from the grapes grown on the different soil types. The total acid content of wine is influenced by maturity level and berry density and was greater in the wines made from the grapes grown on the irrigation silting soil relative to the sierozem and aeolian soils. The wine made from grapes grown on the aeolian soil had the highest anthocyanin content, followed by the wines made from the grapes grown on the sierozem and irrigation silting soils. The anthocyanin content was significantly lower in the wines from the irrigation silting soils relative to the other two soil types.

**Table 4:** Wine composition analysis that produced on three soil types.

Treatment	Alcohol %vol	Residual sugar g L^-1^	Total acid g L^-1^	Dry extract g L^-1^	Chroma	Tone	pH	Anthocyanins mg L^-1^	Total phenols mg L^-1^	Tannins mg L^-1^
Aeolian soil	12.29±0.02a	2.53±0.02a	6.17±0.11b	23.35±0.01a	8.23±0.04a	0.73±0.01a	3.72±0.02a	245.50±1.21a	1912.50±45.36b	2034.75±25.46b
Sierozem	12.48±0.06a	2.52±0.01a	6.24±0.06b	23.30±0.02a	8.18±0.02a	0.75±0.01a	3.72±0.02a	243.55±1.00a	2088.00±38.42a	2172.50±30.29a
Irrigation silting soil	11.40±0.01b	2.51±0.01a	6.66±0.09a	23.25±0.01a	6.06±0.63b	0.55±0.03b	3.71±0.01a	189.43±1.35b	1654.50±41.37c	1773.75±56.87c

Note: Multiple comparisons were conducted among different levels of the same factor in one column (p<0.05).

Soil types played an important role in the tannin and total phenol contents of the grapes. The wine made from grapes from the sierozem soil had the highest tannin and total phenol contents, followed by the aeolian soil and the irrigation silting soil, the latter of which was significantly lower than the other two soil types.

Similar to the phenolic compound results, the chromas of the wines made from the grapes grown on the aeolian and sierozem soils were not significantly different. The wines from the aeolian and sierozem soils both had chromas of approximately 8.20, which was significantly greater than that of the wine from the irrigation silting soil (Table 4). The wines that originated from the aeolian and sierozem soils had nearly the same tone, which were significantly higher than that of the wine originating from the silting soil (Table 4).

## Discussion

Soil composition as a determining factor for the sensory profile of a wine, the types and texture will influence the root systems and the soil water-holding capacity and mineral composition [31,32]. In addition, it can influence the sensory attributes of wine [13,33]. Light loam soils with coarse and fine sands are beneficial for improving grape quality. The photosynthetic rate and fruit sugar and anthocyanin contents of grapes grown on stony sandy loam soils are high. However, the tannin contents of grapes grown on aeolian soils are greater, stickier soil particles are less beneficial for grape composition [3,22,34]. Here, the moderate air and water permeability of the aeolian and sierozem soils resulted in the accumulation of high anthocyanin concentrations and a small berry size. The wine grapes from the irrigation silting soil had tight clusters and relatively high 100-berry weights, which were significantly greater than those of the grapes from the aeolian and sierozem soils.

Sugar, organic acid, phenolic compounds, anthocyanin and aroma substances are all important for wine composition, with sugar being the most important substance. Grapes with higher sugar contents can produce full-bodied wines [31,35]. Thus, our results indicated that the sugar contents of the berries increased rapidly during the maturation period, whereas the acidity decreased before remaining stable (Fig. 2b). The sugar contents and sugar acid ratios of the grapes from the aeolian and sierozem soils were greater than those from the irrigation silting soil. However, the total acid content in the former soils was lower than that of the irrigation silting soil. Berry acidity mainly decreased due to berry respiration, in which the organic acids were transformed into sugars [8,33,36]. Berry acidity not only influences wine flavor but also the fermentation process. The sugar to acid ratio was greater in the wine grapes from the aeolian and sierozem soils relative to the irrigation silting soil.

Some differences in the various phenolic compounds were observed in the regional wines, which suggested that the accumulation of phenolic compounds in grape berries is strongly affected by soil type [10,37–39]. The anthocyanin content, the total phenol content and the tannin contents in the grapes from the aeolian and sierozem soils were greater than in the grapes from the irrigation silting soil. In addition, the aeolian soil provided favorable conditions for the formation of anthocyanin and aroma substances, and the sierozem soil provided favorable conditions for the formation of tannin and phenolic compounds in the grapes, which corresponded with previous results [3,22].

Soil texture had the most wide-reaching influence on the wines from the various sites. However, consistency among vintages was not observed [40,41]. Zones with soils with high clay textures appeared to produce wines with more earthy and citrus aromas, whereas zones with sandy soils produce wines with floral and melon aromas and flavors [33]. It is difficult to discern the impacts of soil texture on wine composition. In clay zones, heavier berries are produced with slightly delayed fruit maturity, lower berry Brix values, higher acidic contents and lower pH values [33]. These previous findings are consistent with our study: the total acid content of the wine grapes from the irrigation silting soil were significantly higher than those of the wine grapes from the sierozem and aeolian soils, and the pH values of the wine grapes from the aeolian soil were greater than those of the wine grapes from sierozem and irrigation silting soils.

At the same grape-ripening level, soil may affect several wine characteristics. Wines from poorer soils with higher coarse fractions exhibit higher total phenolic contents and color intensity but lower resveratrol concentrations [2,41]. The degree of oxidation and composition can be determined based on the wine chroma and tone. The wine made from the grapes grown on the sierozem soil had the highest tannin and total phenol contents, and the wine made from the grapes grown on the irrigation silting soil had the lowest levels. The difference between the chromas of the wines made of the grapes from the aeolian and sierozem soils was not significant. The chroma values of the wines resulting from the aeolian and sierozem soils were significantly greater than the chroma resulting from the irrigation silting soil (Table 4). Furthermore, the wines made from the grapes from the aeolian and sierozem soils had similar tone values, which were significantly higher than that from the wine made from the irrigation silting soil grapes (Table 4). Thus, the wine made from the grapes grown on the sierozem soil had better chroma. If the anthocyanin and tannin contents are high, the tone of the wine will be rich; otherwise, the tone of the wine will be weak.

Different soil types resulted in significant differences in the total sugar and total acid contents of the grapes and, to some extent, the anthocyanin, tannin, phenolic and aroma contents in the berry skin. The grapes from the aeolian soil have small berry size and loose fruit clusters. The skin color was nicer and the skin fruit ratio was higher for the grapes from the sierozem soil. Therefore, the sugar and anthocyanin contents and the color density were greater, which resulted in better tasting and looking wine with a high tannin content. However, the composition of the wine made from the grapes grown on the irrigation silting soil was comparatively lower.

## Conclusions

The grapes grown on the sierozem soil that matured early had high sugar and anthocyanin contents. Consequently, these grapes produced wine with better chroma. Furthermore, the grapes grown on the sierozem soil that matured during a moderate period had medium tannin contents and low acidity. In general, the sierozem soil was favorable for the formation of tannins and phenolic compounds, which influenced the mouthfeel and composition of the wine. The grapes grown on the irrigation silting soil had high acidity and lowest contents of other investigated substances.
